# P064. 12 years of Master Degree in Headache Medicine at Sapienza University of Rome

**DOI:** 10.1186/1129-2377-16-S1-A187

**Published:** 2015-09-28

**Authors:** Noemi Lala, Paolo Martelletti

**Affiliations:** Department of Clinical and Molecular Medicine, Sapienza University, Rome, Italy

The Master in Headache Medicine has accomplished its 12^th^ cycle of life [1]. At the time of its activation we acknowledged the necessity of an academic education thought to support the growth of a subspecialty included in major disciplines like neurology, internal medicine, emergency medicine, pharmacology and pain medicine. The challenge then was to reunite multidisciplinary skills in a common formative path recognized by National Health Systems [2]. The internationalization of the Faculty, the endorsement of the European Headache Federation, the educational arm of Lifting The Burden - The Global Campaign against Headache and the tight collaboration with the European Federation of Pain - Joint Campaign against Headache has allowed to complete an innovative project that has been heavily criticized for its uncertain utility in a contest where this educational strategy was considered only a redundant subspecialty [3, 4]. The educational offer over time included frontal lectures, clinical seminar with tutorial support and mid-term evaluations available on the Moodle platform. The attendance in the outpatient and daily hospitalization wards of a University Hospital with a solid national experience avant-garde on these diseases completed the educational frame. Foreign students could benefit from distance e-learning platform and bursaries for those coming from developing countries.

The qualified international profile of the Faculty guaranteed the excellence of the formative offer.

Today, after 12 years of academic education, Sapienza University of Rome has released 119 master degree diplomas in headache medicine to as many students coming from all the six WHO geographical areas: Africa, Americas, Europe, Eastern Mediterranean, South-East Asia, Western Pacific.

The Train-The-Trainers model, a top-down educational activity, has accomplished its path allowing all the master degree physicians to teach headache medicine locally in favour of GPs. The same Sapienza distance e-learning platform served also as source for this second level of education.

This experience has shown to be fruitful in a mid-long term and particularly useful in geographical areas with a low presence of area experts. After more than 10 years the received feedbacks show the efficacy of this educational spreading model thought to reach the control of headache disorders now ranked as third most disabling disease in the world [5].
